# Ultra-processed foods as a possible culprit for the rising prevalence of inflammatory bowel diseases

**DOI:** 10.3389/fmed.2022.1058373

**Published:** 2022-11-07

**Authors:** Eva Vissers, Judith Wellens, João Sabino

**Affiliations:** ^1^Department of Chronic Diseases, Metabolism and Aging, Translational Research Center for Gastrointestinal Disorders (TARGID), KU Leuven, Leuven, Belgium; ^2^Department of Gastroenterology and Hepatology, University Hospitals Leuven, Leuven, Belgium

**Keywords:** inflammatory bowel diseases, ultra-processed foods, intestinal barrier, diet, food additives

## Abstract

Inflammatory bowel diseases (IBD) are chronic inflammatory disorders of the gastrointestinal tract, and the exact pathogenesis is still unclear. It is believed that IBD develops in response to a complex interaction between the microbiota, environmental factors, and the immune system, in genetically predisposed individuals. Identifying these environmental factors will offer more insight in the development of the disease, and reveal new therapeutic targets for IBD patients. One of the environmental factors that has gained more interest over the last years is our diet. The prevalence of IBD has increased significantly and this increase is thought to be associated with a ‘Western diet', characterized by high intake of fats, added sugar, meat, and ultra-processed foods (UPFs). The UPFs now account for almost 50% of the energy intake in Westernized countries and are therefore an important characteristic of this Western diet. UPFs are characterized by higher amounts of salt, fat, sugar and the presence of different food additives. Epidemiological studies have found associations between UPF intake and a range of non-communicable diseases, including inflammatory bowel disease (IBD). Preclinical and clinical evidence suggest that non-nutritive ingredients and additives, present in UPFs, can negatively affect different components of the intestinal barrier, such as the microbiota, the mucus layer, the epithelium, and the immune cells in the lamina propria. Disruption of this barrier can cause the immune system to encounter an increased bacterial exposure, leading to an aberrant immune response. In this article, the available evidence on the possible role of UPFs and their components in the increasing incidence and prevalence of IBD is reviewed. These findings can be translated to the clinic and may be helpful to consider when giving dietary advice to IBD patients. A better understanding of the role of UPFs may lead to less restrictive diets for patients with IBD, hence increasing the dietary compliance and efficacy of exclusion diets.

## Introduction

Food systems are changing globally and typically encompass a decrease in home cooking and an increased consumption of pre-prepared dishes, fast-food, and ultra-processed food products (UPFs) (1, 2). UPFs can be defined as ready-to-consume products, that consist of a combination of substances derived from foods and food additives, and are formed through several industrial processes. They usually contain high amounts of sugar, saturated fats, and salt, and low amounts of protein, fibers, vitamins and minerals (1, 3). Examples of UPFs are sweet or savory snacks, soft drinks, processed meat products, packaged breads, and pastries (4). In the past decades, an increasing amount of UPF servings became present in the overall diet (2). UPFs now account for more than 50% of the energy intake in Westernized countries, including the United Kingdom, the United States, Australia and Canada, and for about 30% in middle-income countries, such as Brazil, India, and South Africa, with sales that continue to grow (3–5). The high consumption of these UPFs results in a low nutritional quality of the overall diet (1). In addition, UPFs contain non-nutritional ingredients and additives such as preservatives, stabilizers and thickeners, emulsifiers, artificial sweeteners, and colorants.

The consumption UPFs has been associated with many non-communicable diseases including obesity, cancer, cardiovascular diseases, and the metabolic syndrome (6, 7). High UPF consumption has also been associated with inflammatory bowel diseases (IBD), including Crohn's disease (CD) and ulcerative colitis (UC) (8). IBD are chronic inflammatory conditions of the gastrointestinal tract (9, 10). The pathogenesis of the disease is still unclear but it is thought that IBD develops in genetically predisposed individuals that are exposed to a complex interaction between the microbiota, the immune system and environmental factors (11). Frequently cited environmental factors to have been associated with IBD, include smoking, the use of antibiotics, a high level of hygiene, and components of our diet (12, 13). Nevertheless, any specific dietary components that might trigger or worsen IBD are yet to be fully characterized.

The incidence and prevalence of IBD are increasing rapidly worldwide, stressing its global impact and importance (14, 15). To date, the highest prevalence is still found in Westernized regions, such as Europe and North America (14). In these regions, the incidence of IBD has stabilized or is even slightly decreasing (15). However, from the start of the 21st century, IBD incidence and prevalence are increasing remarkably in newly industrialized regions, such as Africa and South America (14, 16). These epidemiological observations cannot be explained by genetic factors alone, suggesting that it is at least partly driven by environmental factors. Epidemiological and (pre)clinical data increasingly point to the Western diet as a possible culprit, which is characterized by an increased consumption of refined sugars, dietary fats, and animal protein, and a decreased consumption of fibers (17). Consumption of these macronutrients have all been associated with IBD risk (18–22). The consumption of fat, refined sugars and animal protein has been associated with higher risk for IBD, whereas high dietary fiber intake has been associated with lower IBD risk. Apart from these macronutrients, recently, the attention has shifted to the non-nutritional components of our diet, the food additives present in UPFs.

In a French prospective cohort study, no association between the risk of IBD and the consumption of UPFs was observed (23). However, only 75 out of 105,832 participants were diagnosed with IBD, which limited the statistical power of the study. Subsequently, in a large prospective cohort study from Narula *et al*., including 116,087 participants from both low, middle and high income countries, a higher intake of UPFs was associated with an increased risk of IBD (8). They also observed an association between the risk of IBD and the consumption of individual types of UPFs, such as processed meat, soft drinks, refined sweetened foods, fried foods, and salty foods and snacks. These results applied for both CD and UC. A larger prospective study, including 245,112 participants, confirmed the association between the risk of CD and the consumption of UPFs, but found no association for UC (24).

Since UPFs are comprised of a large category of food products, include several macronutrient and micronutrient elements, and are characterized by distinct processing and packaging methods, it is important to identify the substances in UPFs that could be responsible for the association between the consumption of UPFs and IBD risk. The possible role of macronutrients in the development of IBD was reviewed elsewhere, and is outside of the scope of this review (25–27). This article reviews how several non-nutritive substances, typically present in UPFs, can affect the intestinal barrier and therefore play a role in the development of IBD in order to explain the above mentioned epidemiological associations. The available preclinical and clinical evidence with their translational potential will be discussed as a promising treatment strategy to tackle bowel inflammation, especially in IBD patients.

## What are ultra-processed foods?

Food processing itself has been used for ages and was necessary in human nutrition and evolution (28). Heating, cutting, drying, fermenting, and salting of food are all processes which were needed to guarantee microbiological safety, the ability to preserve food, accessibility, and affordability of food (7). The processing of foods can be classified according to the NOVA classification, developed by Monteiro and colleagues (1). This classification system divides food products into four groups, based on the nature, extent and purpose of industrial processing they undergo (1, 29). Group 1 contains the unprocessed or minimally processed foods, such as fruits, vegetables, milk, unprocessed meats, and eggs (1). Food products in group 2 are called ‘processed culinary ingredients' (1). These products are substances that are derived from nature or from group 1 products by industrial processes (pressing, drying, refining etc.). Examples of group 2 are oils, sugar, and salt, and these products are meant to be used in combination with other food products. Group 3 comprises the processed foods (1). These processed foods often consist of the combination of two or three ingredients from group 1 and 2 that underwent processes like non-alcoholic fermentation, preservation methods and cooking methods. Examples of group 3 are bread, cheese and bottled vegetables. The last group, group 4, are the UPFs (1). These food products are mostly made from substances derived from foods, and additives, such as soft drinks, pre-packaged meals, processed meat products, and packaged snacks. Different industrial processes are used to combine the usually many ingredients, such as hydrogenation and hydrolyzation, extrusion, and fractioning.

In general, UPFs are energy-dense, rich in refined sugars, fats, salt, and food additives, and low in proteins, fibers, and micronutrients (1). Therefore, the overall nutritional quality of UPFs is lower than of unprocessed foods. The goal of ‘ultra-processing' is to make the foods more palatable, ready-to-consume, highly profitable, and extend their shelf-life (1, 7). Economically, there is a lot of attention for the branding and marketing of these products, often targeted toward children and young adults (1). The combination of these factors makes UPFs more prone to overconsumption.

## The intestinal barrier: An important player in IBD

The gastrointestinal immune system has a complex task. It is responsible for the protection against pathogens that are present in the gut lumen, but on the other hand it has to gain tolerance against commensal micro-organisms and food components (30).

The intestinal barrier fulfills a crucial role in the maintenance of gut homeostasis and consists of different components. First, commensal microbiota prevent colonization of pathogens and produce anti-inflammatory metabolites, such as short-chain fatty acids (SCFA) (31). The composition and/or activity of these micro-organisms are influenced by, among other things, age, genetics, drugs, and diet (32). Next, a mucus layer existing of glycoproteins, mainly mucin 2 proteins (MUC2), acts as a physical and chemical barrier because it prevents bacterial adhesion and contains antimicrobial molecules (33). Underneath the mucus layer, a single layer of epithelial cells, such as enterocytes and to a lesser extent goblet cells, Paneth cells and enteroendocrine cells, are strongly linked to each other due to the presence of tight junction proteins (30). These tight junction complexes are formed by different proteins, such as occludines, zonula occludens proteins and claudins (34). Lastly, right beneath the epithelium lies the lamina propria, in which loads of immune cells are present.

In IBD, the intestinal barrier can be disrupted at different levels. Intestinal dysbiosis has been described in patients with IBD, and it is characterized by a decreased microbial diversity and a shift from beneficial to potentially pathogenic micro-organisms (35). Specifically, the commensal bacteria of the phyla *Firmicutes* and *Bacteroidetes* are depleted in IBD patients, while *Proteobacteria* and *Actinobacteria* are increased (35). Moreover, the production of SCFAs can be impaired in patients with IBD (36). The mucus layer was observed to be thinner in patients with IBD and MUC2 biosynthesis was impaired (33, 37). These defects in the mucus layer can cause commensal or pathogenic bacteria to colonize the mucus and increase the contact between micro-organisms and the epithelial cells (33). It was also shown that patients with IBD have an increased permeability of the intestinal epithelium (38). As a result, micro-organisms or other antigens present in the gut lumen can come in contact with the immune cells in the lamina propria to a greater extent, leading to activation of the immune system and intestinal inflammation.

**Figure 1:**
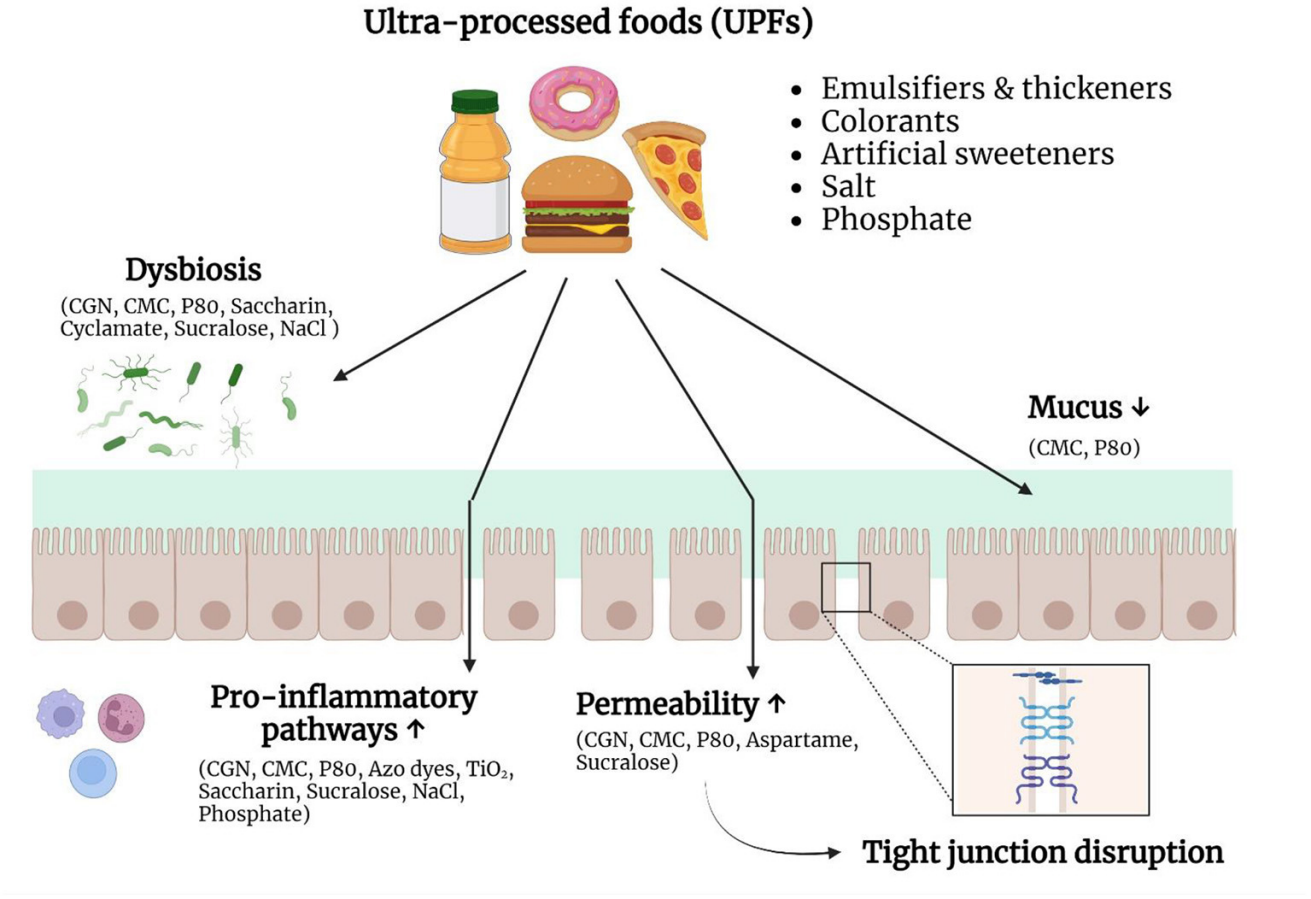
Non-nutritive ingredients and additives in ultra-processed foods (UPFs) can negatively affect the intestinal barrier. UPFs typically consist of a combination of substances derived from foods and food additives. Growing evidence suggests that these non-nutritive ingredients and food additives are able to interact with the intestinal barrier and therefore play a role in the pathogenesis of IBD. Different food additives can induce dysbiosis, stimulate pro-inflammatory pathways, increase the epithelial permeability, or alter the mucus layer. Due to these changes, the immune system encounters an increased bacterial exposure, which can result in chronic intestinal inflammation. CGN, carrageenan; CMC, carboxymethylcellulose; P80, polysorbate-80; NaCl, sodium chloride; TiO_2_, titanium dioxide. Created with Biorender.com.

Although disruption of the intestinal barrier is associated with IBD and other diseases, such as celiac disease and irritable bowel syndrome (IBS), it remains unclear if this is a cause or consequence of the diseases (38). Therefore, it is important to identify substances with the capacity to affect one or more components of the intestinal barrier. Evidence that the non-nutritional components of UPFs can negatively affect the intestinal barrier is growing (Figure 1). They can directly interact with the mucus layer, epithelial cells or immune cells and in this way increase the intestinal permeability and/or promote inflammatory pathways. On the other hand, they can also indirectly affect gut homeostasis, by altering the composition and/or function of the microbiota.

## Impact of UPF components on the intestinal barrier: Preclinical and clinical evidence

### Emulsifiers and thickeners

Emulsifiers are commonly added to UPFs, such as sauces, industrial bread and pastries, and meat substitutes, to increase the stability, shelf-life, and palatability of food products (39, 40). Different studies have shown that emulsifiers can have an effect on each of the components of the intestinal barrier and thereby play a role in the development of IBD (41).

Some commonly used synthetic emulsifiers, such as carrageenan (CGN) and carboxymethylcellulose (CMC), reach the colon in an quasi intact form, and are able to interact with the microbiota (39). Studies have been conducted in which human stool was incubated with food additives, including emulsifiers (42, 43). Incubation with polysorbate-80 (P80) or CGN for 24 hours resulted in an alteration of the microbiota composition and their SCFA-producing activity (42). A study in interleukin (IL)10^−/−^ and Toll-like receptor 5 (TLR5)^−/−^ mice, animal models that spontaneously develop colitis, showed that the ingestion of CMC and P80 causes a shift in the gut microbiota composition to more mucus-degrading bacteria and a decrease of the microbial diversity (44). The fecal concentration of flagellin, a bacterial protein that is known to have a pro-inflammatory potential, did increase after emulsifier ingestion (44).

Furthermore, emulsifiers can alter the amount of mucus and the structure of the mucus layer in the gastrointestinal tract. When porcine small intestinal mucus was incubated *in vitro* with two common emulsifiers, CMC and P80, the mesh structure was affected (45). In murine models, the administration of CMC and P80 resulted in thinning of the mucus layer (44). This damaging effect of emulsifiers on the mucus layer seemed to be driven by the microbiota, as this effect was not observed in germ-free (GF) mice. However, upon exposure to microbiota from emulsifier-treated mice, a thinner mucus layer was observed (44).

The permeability of the intestinal epithelium can also be affected by dietary emulsifiers (46). It is hypothesized that when this happens, the microbiota in the intestinal lumen can interact to a higher degree with the immune cells in the lamina propria, resulting in activation of the immune response. Interestingly, an increased intestinal permeability has already been observed in patients with IBD (38). An *in vitro* study from Roberts *et al*. showed that the emulsifier P80 increased the translocation of *Escherichia coli* over M-cells and thus increased the transcellular permeability (47). Other *in vitro* studies observed that exposure of intestinal cell cultures to emulsifiers, such as P80 and CGN, resulted in an altered expression or a dislocation of tight junction proteins (48, 49). In mice, ingestion of CMC and P80 caused an increase in the intestinal permeability, both in wild-type (WT) as in genetically predisposed mice (44).

Lastly, emulsifiers could directly interact with the immune cells in the lamina propria, and in this way influence the mucosal immune response. *In vitro* studies showed that CGN can activate the pro-inflammatory NF-κB pathway, resulting in the production of pro-inflammatory cytokines, such as IL-8 (50). Later, TLR4 was indicated as the responsible receptor for this effect (51). Chassaing et al. observed in their study that the administration of CMC and P80 increased the incidence of colitis in IL10^−/−^ and TLR5^−/−^ mice, that are predisposed for developing intestinal inflammation (44). In WT mice, the emulsifiers did not cause severe colitis, but signs of low-grade inflammation were observed. These harmful effects were again attributed to the microbiota (44).

To date, only a handful of clinical trials have been conducted with emulsifiers in the context of IBD. Firstly, a study evaluated the feasibility and adherence to a 14-day low-emulsifier diet in 20 participants with CD (52). Following the low emulsifier diet led to significant improvement in symptoms, disease control, and quality of life. These results are promising, but comparing this low-emulsifier diet with a control diet in a larger clinical trial is necessary to rule out a placebo-effect. Another dietary intervention feasibility study advised 28 adults with UC to increase their intake of fibers, restrict total and sulfur-containing proteins, and avoid specific food additives, including CGN, for 8 weeks (53). The dietary intervention achieved clinical improvement in 46% of the participants, endoscopic improvement in 36% of the participants, and improvement in quality of life in 43% of the participants. Bhattacharyya et al. studied if CGN could contribute to the risk of relapse in UC patients in remission (54). All participants followed a CGN-free diet and the intervention group consumed 200 mg CGN per day in the form of CGN-capsules, which were compared to placebo capsules in the control group. This study concluded that the consumption of CGN contributed to exacerbations of UC and increased the risk of relapse. In a randomized controlled-feeding study with 16 healthy volunteers from Chassaing et al. an emulsifier-free diet was compared with CMC supplementation for 11 days (55). They observed that the consumption of CMC led to a decrease in microbial diversity and a reduction of SCFA production. In two of the seven participants in the intervention group, they observed that the intestinal mucus layer had become thinner, as evidenced by encroachment of the microbiota.

### Food colorants: Azo dyes

Synthetic food colorants are widespread in a Western diet and aim to enhance the appearance of food products and drinks (56). Products containing these food colorants are candies, dairy products and soft drinks. Little is known about the possible role of food dyes in the development of IBD. According to the World Health Organization (WHO), food colorants Red 40 and Yellow 6 are considered safe for human consumption (57). However, a recent landmark study from He et al. showed that the azo dyes Red 40 and Yellow 6 were capable of triggering IBD-like colitis in mice with an overexpression of IL-23, but not in control mice (58). The responsible substance for this induction of colitis was shown to be a metabolite derived from the reduction of the azo group by specific commensal bacteria, such as *Bacteroides ovatus* and *Enterococcus faecalis* (58, 59). Dysregulation of IL-23 expression was necessary for Red 40 to induce colitis, and dependent on the production of interferon (IFN)-γ by effector CD4+ T-cells. Since the IL-23 pathway is known to be linked with IBD and successfully targeted in IBD treatments (namely ustekinumab and rizankizumab), these azo dyes could aggravate inflammation or elicit flares in patients with IBD (58, 60).

### Food colorants: Titanium dioxide

Titanium dioxide (TiO_2_) is used as a food additive because of its whitening and brightening properties and is referred to as E171 in the European Union (61). TiO_2_ is mostly used in confectionary, candies and chewing gum, white sauces, and icing, but also in toothpaste and pharmaceuticals (61). Exposure of TiO_2_ is estimated to be around 0.2–0.7 mg/kg/day in the United States (61). Food-grade TiO_2_ consists of a broad size range of TiO_2_ particles, with up to one third of them being nanoparticles (<100 nm) (61, 62). Studies suggest that TiO_2_ nanoparticles can accumulate in the cells of the Peyer's patches (PP) in the gut and in this way contribute to intestinal inflammation.

In a rat model, food-grade TiO_2_ was orally administered to rats for 7 days (63). TiO_2_ particles did accumulate in the PP, as well as in the colonic mucosa and the liver. The PP are rich in immune cells, and TiO_2_ administration resulted in a decrease in dendritic cells and a decline in T-cell proliferation. Further, when immune cells were isolated from the PP and stimulated *ex vivo* with TiO_2_, the secretion of IFN-γ was decreased. There were no changes in intestinal permeability, neutrophil infiltration or changes in cytokine content in the mucosa after 1 week of TiO_2_ administration. However, after chronic administration for 100 days, cytokine levels of IL-6, Tumor necrosis factor (TNF)-α, IL-8 an IL-10 were elevated in the colonic mucosa of the rats, reflecting the development of low-grade inflammation.

A pilot study in 20 patients evaluated if a reduction of microparticles in the diet could improve the symptoms of CD (64). In this study, it was observed that a diet low in microparticles improved the symptoms and disease activity, determined by the Crohn's Disease Activity Index, already after 1 month. However, only 20 participants completed the study, making the study underpowered. In a larger, fully powered clinical trial from the same research group, no effect of a diet low in microparticles was observed (65). This means there is no evidence yet for reducing microparticle intake in active CD in clinical practice.

### Artificial sweeteners

The consumption of artificial sweeteners, such as sucralose, aspartame, and saccharin, has increased over the past decades (66). The main reason for the extensive use of artificial sweeteners is to reduce the caloric content of foods and beverages. Indeed, it is known that excessive sugar consumption leads to adverse health effects, such as obesity and metabolic syndrome (67). Besides, excessive consumption of simple carbohydrates has been shown to alter the microbial composition and promote colitis in mice (68). Artificial sweeteners have a higher sweetening intensity than sugar, but contain significantly less calories and are therefore also called “low-calorie sweeteners” or “non-caloric sweeteners.”

*In vitro* studies have showed that artificial sweeteners can interact with different components of the intestinal barrier and evoke or promote intestinal inflammation. A study using Caco-2 cells, a neoplastic human intestinal epithelial cell line, showed that high concentrations of aspartame and saccharine could induce apoptosis and cell death (69). At lower concentrations, the sweeteners increased the permeability of the epithelial barrier. A down-regulation of the tight junction protein claudin 3 was observed. Further, aspartame induced the production of reactive oxygen species (ROS), which caused the increased permeability and claudin 3 internalization. In a study of Vamanu et al., the sweeteners saccharin and cyclamate were able to cause a decrease in the total number of microorganisms and lowered the production of SCFAs (70).

In animal studies, saccharin induced dysbiosis that was linked to alterations in glucose tolerance (71). Furthermore, in another murine study, mice receiving saccharin in their drinking water for 6 months had alterations in the gut microbiota with an increase in inflammatory metabolites (72). Saccharin was also able to induce microbiota-driven liver inflammation, with elevated expression of inducible nitrogen oxide synthase (iNOS) and TNF-α (72). The same study was repeated with sucralose, and sucralose ingestion resulted in an enhancement of bacterial pro-inflammatory gene expression, such as lipopolysaccharide (LPS) synthesis and toxin production (73). Sucralose seems to promote inflammation leading to exacerbation of dextran sulfate sodium (DSS) -induced colitis in mice (74). This effect is associated with changes in the microbiota and intestinal barrier, and an enhancement of inflammatory cytokines and pathways. In contrast, a more recent study in mice observed that saccharin supplementation improved DSS-induced colitis activity, by decreasing the intestinal bacteria count (75).

There are only a few human trials published on artificial sweeteners and intestinal health. First, Suez et al. observed that the consumption of saccharin for 1 week caused a poorer glycemic response in four out of seven healthy adults, which was accompanied by compositional changes of the microbiota (71). Another study in humans did not observe a difference in bacterial abundance between consumers of aspartame or acesulfame-K and non-consumers (76). Lastly, in a small randomized double-blinded crossover clinical trial, the effect of sucralose and aspartame consumption on gut microbiota composition was evaluated (77). After consumption of both sweeteners for 14 days, the microbiota community structure did not change significantly. There were also no changes in fecal SCFAs observed. This shows that the preclinical data always warrant translational human trials to make conclusions.

### Salt

UPFs typically contain a much higher salt content than home-cooked meals. Dietary salt or sodium chloride (NaCl) consists of 40% sodium and 60% chloride (78). The WHO recommends to not consume more than 5 g of salt per day, but these recommendations are often exceeded (79). The consumption of salt has been increasing over time and the link between an excessive salt intake and hypertension and other cardiovascular diseases is well-known (80, 81). However, both *in vitro* and *in vivo* studies suggest that excessive salt intake can also modulate the immune system, and in this way be involved in the development of IBD (78).

In *in vitro studies*, an increase in the concentration of NaCl could induce pro-inflammatory cytokine production by mononuclear cells, derived from the intestinal lamina propria (82). Excess sodium could also stimulate murine dendritic cells to increase the production of IL-1β, which then promoted the production of IL-17A and IFN-γ, two pro-inflammatory cytokines, by T-cells (83). Furthermore, NaCl was capable of inhibiting forkhead box protein 3 (Foxp3+) regulatory T-cells, which normally warrant self-tolerance, and activating the pro-inflammatory Th17 cells (84, 85).

The effect of dietary sodium chloride on the gut has also been investigated, using different animal colitis models (78). A murine study demonstrated that high salt intake can affect the gut microbiome, specifically decreasing the abundance of *Lactobacillus murinus* (86). Another murine study also showed that a high salt diet (HSD) caused alterations in the composition and function of the intestinal microbiota in mice (87). The HSD led to a reduction in *Lactobacillus* species and butyrate production. Moreover, the expression of pro-inflammatory genes was enhanced, and the mice on a HSD developed more severe DSS-induced colitis. Interestingly, no harmful effects of the HSD in GF mice were observed. When microbiota from HSD-fed mice was later transferred to GF mice, they did develop colitis, suggesting that the microbial alterations are the driving factor for the pro-inflammatory effect of excessive sodium intake (87). In a study of Tubbs *et al*. mice were fed with either a low salt diet (LSD) or a HSD, with the latter having a comparable sodium content as a typical Western diet (88). The mice on a HSD had significantly higher fecal concentrations of sodium. In IL10^−/−^ mice, the HSD exacerbated colitis compared with the LSD (88). This was reflected in an increased expression of pro-inflammatory cytokines in the colonic tissue, such as TNF and IL-23. However, in WT mice there was no difference in the degree of inflammation between both diets, meaning that the high salt intake itself was not enough to cause intestinal inflammation without a predisposing genetic background.

Studies delivering human data on the immunological effect of a diet high in salt are limited. In a small randomized controlled trial in six heathy male volunteers, switching from a HSD (12 g/day) to a LSD (6 g/day) led to a reduction in the number of blood monocytes and pro-inflammatory cytokines, such as IL-6 and IL-23, and an enhanced production of the anti-inflammatory cytokine IL-10 (89). From these results, Yi et al. concluded that the consumption of a HSD may potentiate extensive immune responses (89). In another small uncontrolled human study, 12 healthy adults received 600 mg of NaCl on top of their normal diet for 2 weeks (86). They observed that this high salt consumption led to an increase in CD4+ T-cells, Th17 cells, and an increased production of the pro-inflammatory cytokines IL-17A and TNF-α. The high salt consumption also resulted in an alteration of the intestinal microbiome composition, with a reduction in *Lactobacillus* species. Despite that these results all show that a HSD can have a systemic pro-inflammatory effect, studies in humans that are specifically related to IBD are still lacking.

Contradictory to the above mentioned *in vitro* and *in vivo* studies, in the observational study of Narula *et al*., an association between the risk of IBD and salt consumption was not observed (8). Thus, more human research on this topic is necessary to evaluate if decreasing the salt consumption could induce or keep remission, or improve the symptoms in IBD patients.

### Phosphate

Inorganic phosphate is regularly present in UPFs, due to the presence of phosphate in different types of food additives, such as emulsifiers (e.g., sodium phosphate), pH stabilizers (e.g., phosphoric acid), and nutritional supplements (e.g., dicalcium phosphate) (90). To our knowledge, only one study, comprising both *in vitro* and *in vivo* experiments, was conducted where the effects of dietary phosphate on experimental colitis was evaluated (91). *In vitro*, RAW264.7 macrophages produced more ROS and pro-inflammatory cytokines after high phosphate loading. Phosphate-mediated induction of ROS can activate the NF-κB pathway, a pro-inflammatory pathway which is involved in IBD, and in this way promote the production of pro-inflammatory cytokines. *In vivo*, dietary phosphate exacerbated experimental colitis, induced by DSS, in a dose-dependent manner in rats. The rats on a high phosphate diet had a shorter colon length, more severe colonic epithelial damage, a reduced number of mucus producing goblet cells, and myeloperoxidase activity and the expression of pro-inflammatory cytokines were upregulated in the inflamed colon. The underlying mechanisms was proposed to be the capability of phosphate to stimulate the NF-κB pathway, a pro-inflammatory pathway which is involved in IBD. These results suggest that excessive dietary phosphate intake can enhance intestinal inflammation, but further epidemiological and clinical studies are necessary to confirm this.

## Discussion

Currently, the standard therapies for IBD, such as corticosteroids and monoclonal antibodies, are targeting the immune system (92). However, a large proportion of patients loses therapy response over time and therapeutic goals are not always met (93). As discussed earlier, other components of the intestinal barrier, could also be involved in the development and disease course of IBD. Concurrently targeting the different components of the intestinal barrier, for example by combining classic therapies with dietary changes, might offer a solution. Diet is a promising environmental risk factor for disease onset and severity of IBD, and excessive intake of specific macronutrients present in UPFs, such as saturated fats, simple carbohydrates, and animal proteins, is associated with IBD (94). Recently, also the non-nutritive components of UPFs have gained interest. Both preclinical and clinical studies, summarized in this article, have shown possible mechanisms by which food additives can negatively affect gut homeostasis.

Therefore, dietary changes are a promising strategy to be incorporated into the therapeutic strategies for IBD (94). Epidemiologic studies have shown associations between macro- and micronutrients and IBD risk (94). Additionally, more than half of IBD patients indicate to have changed their diet in response to their symptoms (95). The most studied dietary intervention for CD is the use of exclusive enteral nutrition (EEN). EEN is used in the first-line treatment of pediatric patients with active mild-to-moderate CD and has proven to result in clinical and endoscopic remission (96). However, the results among adults are more variable, which is reflected in its limited use. The exact working mechanisms of EEN are not clear yet, but it is thought that the positive effects of EEN can be attributed to changes in the microbiota (97). Nevertheless, EEN is not well-tolerated by every patient and the unpalatability and social limitations cause difficulties with compliance. Therefore, an anti-inflammatory whole-food diet that mimics the composition of EEN was developed, which is called the Crohn's Disease Treatment With Eating Diet (CD-TREAT) (98). In this diet, emulsifying agents, processed meats, and animal fat are excluded and protein, vitamins, minerals and fiber content are increased (94). These dietary changes are able to establish the same microbial alterations as EEN, decrease gut inflammation, and induce clinical remission in patients with active CD (98, 99).

Much more exclusion diets have been designed over the years. These exclusion diets are based on the avoidance of certain dietary components that are believed to affect components of the intestinal barrier. Remarkably, all of these diets exclude UPFs and specific groups of food additives, such as emulsifiers (100). The Crohn Disease Exclusion Diet (CDED) eliminates processed meats, products containing emulsifiers, and canned goods (101). The Autoimmune Protocol Diet (AIP) excludes coffee, alcohol, oils, refined sugars and food additives (102). Because food additives seem to have a negative impact on the intestinal barrier, a recent dietary guidance from the International Organization for the study of IBD, suggested a decreased intake of food additives for patients with IBD (103).

The preclinical and limited clinical data, summarized in this article, suggest that lowering UPF intake could be a beneficial dietary strategy for IBD patients. However, larger human trials are still lacking. Besides, in all the above-mentioned studies, food additives are tested as a single ingredient, whereas in most food products, a combination of different additives which may interact with one another is added. It is possible that two or more non-nutritive components, present in one UPF, can interact with different parts of the intestinal barrier, and enhance each other's detrimental effects. If for instance the mucus layer is affected by an emulsifier, other additives are able to interact with the epithelial cells to a higher extend. Hence, studies with multi-ingredient products are necessary to evaluate the effects of the combination of additives on intestinal health (104). Moreover, effects of food additives on intestinal health can depend on the microbiome and the individual metabolomic profile. In a randomized clinical trial from Suez et al., 120 healthy adults, who consumed no artificial sweeteners in their regular diet, were given supplements with either saccharin, sucralose, aspartame, or stevia sachets for 14 days (105). All supplements contained glucose as a bulking agent, so a fifth group was given glucose supplements as control, and a sixth group did not receive any supplement. Saccharin and sucralose significantly elevated the glycemic response, compared to glucose. The microbiome composition was also altered after supplementation with saccharin and sucralose and all the sweeteners had an effect on microbial functions. Next, stool microbiome of the participants was transplanted into GF mice. Mice receiving stool from any artificial sweetener supplemented group, developed an elevated glycemic response, but this was not observed when mice received stool from the glucose or non-supplemented group. These results show that food additives, such as artificial sweeteners, can exert an effect on overall health by inducing changes in the microbiome composition and/or function.

Finally, there is a relationship between the consumption of UPFs and the lack of home food preparation skills (106). Encouraging IBD patients to gain these skills or offering tips to increase home cooking could help to reduce UPF consumption (106). This also highlights the importance of dieticians and the incorporation of dietary advice in the management of IBD. Guiding the general population to increase their home cooking could in turn be an opportunity to dampen the rising prevalence of IBD.

## Conclusions

Preclinical studies show that several non-nutritive components of UPFs can negatively affect the intestinal barrier. They can induce dysbiosis, negatively affect the mucus layer, increase the permeability of the intestinal epithelium, or directly interact with the immune system. Based on these results, a diet low in UPFs could have the potential to induce remission or control symptoms in patients with IBD. However, human data are still limited and nutritional trials are necessary to evaluate the clinical potential of new dietary therapies for IBD patients. In the meanwhile, it is sensible to advise our patients with IBD to reduce the intake of UPFs.

## Author contributions

EV performed the literature search and wrote the paper. JW and JS both revised the paper. All authors approved submitting the final manuscript.

## Conflict of interest

Author JS is a Senior Clinical Investigator of the Research Foundation Flanders (FWO), Belgium. He received speaker's fees from Pfizer, Abbvie, Ferring, Falk, Takeda, Janssen, and Fresenius; consultancy fees from Janssen, Ferring, Fresenius, Abbvie, Galapagos, Celltrion, Pharmacosmos, and Pharmanovia; and research support from Galapagos and Viatris. The remaining authors declare that the research was conducted in the absence of any commercial or financial relationships that could be construed as a potential conflict of interest.

## Publisher's note

All claims expressed in this article are solely those of the authors and do not necessarily represent those of their affiliated organizations, or those of the publisher, the editors and the reviewers. Any product that may be evaluated in this article, or claim that may be made by its manufacturer, is not guaranteed or endorsed by the publisher.
